# Supplementary material for: Asynchronous magnetic resonance elastography: Shear wave speed reconstruction using noise correlation of incoherent waves
Source: Magn Reson Med. 2022 Oct 27;89(3):990–1001. doi: 10.1002/mrm.29502 (PMC9792433; doi:10.1002/mrm.29502)
Supplement: Supplementary file 1 — DATA S1 MATLAB code used to generate the numerical simulations of Figure 1 [file MRM-89-990-s002.zip › k-Wave/helpfiles/getkWavePath.html]

getkWavePath :: Functions (k-Wave)


# getkWavePath

Return pathname to the k-Wave Toolbox.

## Syntax

```
path = getkWavePath()
path = getkWavePath(folder_name)
```

## Description

`getkWavePath` returns the full pathname to the root directory in the k-Wave Toolbox using the slash direction native to the users operating system. If a `folder_name` string is provided, this is appended to the pathname. Note, `folder_name` is not checked for existence, the string is simply appended to the pathname with a trailing slash.

## Optional Inputs

|  |  |
| --- | --- |
| `folder_name` | folder name string to append to the pathname |

## Outputs

|  |  |
| --- | --- |
| `path` | full pathname to the root directory in the k-Wave Toolbox |
